# Increased Homotopic Connectivity in the Prefrontal Cortex Modulated by Olanzapine Predicts Therapeutic Efficacy in Patients with Schizophrenia

**DOI:** 10.1155/2021/9954547

**Published:** 2021-09-01

**Authors:** Xiaoxiao Shan, Rongyuan Liao, Yangpan Ou, Yudan Ding, Feng Liu, Jindong Chen, Jingping Zhao, Yiqun He, Wenbin Guo

**Affiliations:** ^1^National Clinical Research Center for Mental Disorders and Department of Psychiatry, The Second Xiangya Hospital of Central South University, Changsha, 410011 Hunan, China; ^2^The Second Affiliated Hospital of Xinxiang Medical University, Xinxiang, Henan, China; ^3^Department of Radiology, Tianjin Medical University General Hospital, Tianjin 300000, China; ^4^Department of Psychiatry, The Third People's Hospital of Foshan, Foshan, 528000 Guangdong, China

## Abstract

**Background:**

Previous studies have revealed the abnormalities in homotopic connectivity in schizophrenia. However, the relationship of these deficits to antipsychotic treatment in schizophrenia remains unclear. This study explored the effects of antipsychotic therapy on brain homotopic connectivity and whether the homotopic connectivity of these regions might predict individual treatment response in schizophrenic patients.

**Methods:**

A total of 21 schizophrenic patients and 20 healthy controls were scanned by the resting-state functional magnetic resonance imaging. The patients received olanzapine treatment and were scanned at two time points. Voxel-mirrored homotopic connectivity (VMHC) and pattern classification techniques were applied to analyze the imaging data.

**Results:**

Schizophrenic patients presented significantly decreased VMHC in the temporal and inferior frontal gyri, medial prefrontal cortex (MPFC), and motor and low-level sensory processing regions (including the fusiform gyrus and cerebellum lobule VI) relative to healthy controls. The VMHC in the superior/middle MPFC was significantly increased in the patients after eight weeks of treatment. Support vector regression (SVR) analyses revealed that VMHC in the superior/middle MPFC at baseline can predict the symptomatic improvement of the positive and negative syndrome scale after eight weeks of treatment.

**Conclusions:**

This study demonstrated that olanzapine treatment may normalize decreased homotopic connectivity in the superior/middle MPFC in schizophrenic patients. The VMHC in the superior/middle MPFC may predict individual response for antipsychotic therapy. The findings of this study conduce to the comprehension of the therapy effects of antipsychotic medications on homotopic connectivity in schizophrenia.

## 1. Introduction

Schizophrenia, as we know, is a chronic and serious mental illness, and previous studies have shown its interhemispheric deficits [1, 2]. Electroencephalography measurements revealed that schizophrenic patients exhibited abnormalities in the ancipita interhemispheric alpha band consistency among cognitive/activation assignments [3]. The reduction in ancipita redundancy increased in schizophrenic patients [4] suggests decreased interhemispheric cooperation [5].

The human brain displays a homotopic mutual effect that is adjusted through the commissural system, which includes interthalamic adhesions, cerebellar, anterior, and posterior commissures, and corpus callosum [6]. The interhemispheric deficits may be ascribed to structural defects in the commissural system. Previous in vivo imaging and postmortem studies revealed that schizophrenic patients had reduced callosal thickness [7]. The reduced sectional anisotropy of the corpus callosum region in schizophrenic patients suggests interhemispheric hypoconnectivity [8], which may be associated with the prediction of the severity degree of psychosis symptoms [9].

Interhemispheric functional connectivity changes have been revealed in all phases of schizophrenia. For example, reduced homotopic connectivity in the postcentral region and the superior temporal region has been found in early-onset schizophrenic patients, and the interhemispheric synchronization deficits are negatively related to the negative symptom of the positive and negative syndrome scale (PANSS) [1]. Liu et al. revealed decreased homotopic connectivity in the fusiform and precentral gyri, precuneus, and superior temporal gyrus (STG)/insula in adolescent-onset schizophrenia [10]. In addition, our previous studies showed reductions in interhemispheric functional homotopy in the precentral and middle occipital gyri and STG in schizophrenic patients [11] and in the angular and lingual gyri in the impervious siblings of schizophrenic patients [12]. Nevertheless, it remains unknown whether reduced homotopic connectivity of brain regions is linked to clinical symptoms and predicts treatment response.

Many studies have attempted to illustrate the effects of antipsychotic therapy on brain functions [13, 14]. However, their results remain inconsistent due to the diversity of antipsychotic treatments and the heterogeneity of schizophrenia [15]. Several studies have revealed the normalization and denormalization of blood oxygen level related to functional magnetic resonance imaging (fMRI) signals related to drug therapy. Normalization has been found in temporal and frontoparietal networks, sensorimotor circuits, and default-mode network (DMN) after antipsychotic therapy [13, 16–19]. By contrast, denormalization has been revealed in cortical-subcortical areas, including the ventral lateral prefrontal regions, putamen, and caudate [13, 17–20]. Their results indicate that antipsychotic medicines influence cerebral function with inconsistent outcomes due the discreteness of brain regions. Several factors may cause inconsistent findings. First, studies have employed diverse designs and concentrated on diverse respects of cerebral functions and produced diverse outcomes. Hence, resting-state fMRI, which is a standard design for fMRI studies, is effortless to implement and stops confounding factors regarding performance in clinical studies. Second, the inclusion of patients with chronic conditions and medication in many previous studies may have confounded the results because the course of illness and exposure to different antipsychotics may have neurotoxic influences on the brain. Furthermore, the involvement of different antipsychotic agents in the patient sample in some studies may cause differential effects on brain function. Notably, as far as we know, only one study was used to evaluate alterations in functional homotopy associated with antipsychotic therapy in schizophrenia [14]. Thus, the therapy effects of antipsychotic medicine on homotopic connectivity in schizophrenia remain unclear.

Voxel-mirrored homotopic connectivity (VMHC) [21] is a way used to analyze homotopic connectivity between a given voxel in one hemisphere and its mirrored voxel in the opposite hemisphere. VMHC analysis has been extensively used to explore the changes in homotopic connectivity in individuals with diversified psychiatric disorders, including schizophrenia [2, 11, 22], depression [23, 24], somatization disorder [25], and autism [26], as well as the unaffected siblings [12]. However, whether antipsychotic treatment can affect homotopic connectivity remains unknown.

Previous studies have reported that some brain regions may be related to clinical symptoms and can be applied to predict therapy reaction in schizophrenia. For example, the increased basal ganglia volume, reduced connectivity of the anterior cingulate region, and increased volume and abnormal brain activity of the putamen are related to the clinical outcome and the prediction of therapy reaction in schizophrenia [27–32]. In addition, Shafritz et al. revealed that frontal hyperactivity might offer a biomarker to predict therapy reaction through a simple fMRI task in first-episode psychosis [33]. The increased baseline amplitude of low-frequency fluctuation (ALFF) activity of the inferior parietal lobule/left postcentral gyrus might be associated with the prediction of early treatment response in schizophrenia [34]. A review has shown that reduced volume in the prefrontal cortical and medial temporal regions and the networks combining subcortical structures are associated with poor symptoms and function results [35]. In addition, Veena et al. found that the positive connectivity between the cerebellum and the dorsolateral prefrontal region was related to a good therapy reaction to cognitive behavioral treatment for psychosis in schizophrenia [36]. However, how well the previously presented predictive model could be replicated and validated in schizophrenic patients outside of a single study sample remains unknown.

Previous studies have revealed that psychosis might be predicted on the basis of neuroanatomical biomarkers at the individual level by utilizing multivariate pattern recognition approaches, such as support vector regression (SVR) and support vector machine [37–40]. These pattern techniques have been used to discriminate prodromal subjects [37] and schizophrenic patients [38] from healthy controls and to predict responses to electroconvulsive treatment in major depressive disorder (MDD) and schizophrenia [39, 40].

In the present study, 21 schizophrenic patients were enrolled to explore alterations in VMHC associated with the effect of olanzapine treatment and its role in predicting subject treatment response. The fMRI scans and clinical status of schizophrenic patients were acquired at two time points (baseline and eight weeks of treatment). Olanzapine treatment was hypothesized to normalize VMHC abnormalities in schizophrenic patients. Correlations between VMHC alterations and reductions in symptomatic severity were also explored because they might be underlying biomarkers for predicting individual therapeutic response by the SVR analyses.

## 2. Materials and Methods

### 2.1. Participants

A total of 21 right-handed schizophrenic patients aged 18–50 years were enrolled from the Second Affiliated Hospital of Xinxiang Medical University, China, which included both drug-naive patients with first-episode schizophrenia and drug-free relapse patients. For the relapse patients, they had interrupted antipsychotic medication for more than three months. The effects of previous antipsychotics on brain function might be limited. Schizophrenia was diagnosed in accordance with the Structural Clinical Interview for Diagnostic and Statistical Manual of Mental Disorders, Fifth Edition. The total score of the PANSS exceeded 75, and the disease duration since the onset of the illness did not exceed five years. Symptomatic severity was evaluated with PANSS at baseline and after eight weeks of treatment. Cognitive function was evaluated through the Measurement and Treatment Research to Improve Cognition in Schizophrenia Consensus Cognitive Battery, including the Brief Assessment of Cognition in Schizophrenia Symbol Coding Test(BACS-SC); Brief Visuospatial Memory Test-Revised(BVMT-R); Trail Making Test, part A(TMT-A); Hopkins Verbal Learning Test-Revised(HVLT-R); Neuropsychological Assessment Battery-Mazes(NAB-M); Continuous Performance Test-Identical Pairs(CPT-IP); Wechsler Memory Scale Spatial Span(WMS-SS); Mayer-Salovey-Caruso Emotional Intelligence Test(MSCEIT); and Category Fluency-Animal Naming Fluency(CF-ANF). These tests assessed attention/vigilance, processing speed, reasoning, working memory, problem solving, and verbal learning. All patients were prescribed with olanzapine. Olanzapine dosage was gradually increased on the basis of the patient's condition within the first two weeks and kept unchanged until the last fMRI scan. No other antipsychotics were allowed.

Twenty healthy controls unrelated to the patients were enrolled from the local community. The age and sex ratio of healthy controls and patients were matched. The Structured Clinical Interview for DSM-IV (nonpatient version) was employed to screen healthy controls. The healthy controls were ruled out if they suffered from any medical disorders and neurological disease, psychosis symptoms, or substance abuse. Potential controls were also ruled out if they had a first-degree relative who had a history of psychiatric illness.

The exclusion criteria for all subjects were as follows: (1) any severe physical illness, such as cardiovascular, liver, and kidney illnesses; (2) any neuropsychiatric disorder; (3) seizures; (4) any traumatic brain injury; (5) drug or alcohol addiction; (6) serious impulsive behavior; (7) a history of olanzapine treatment that was ineffective or tolerable; (8) contraindications for the MRI scan; and (9) pregnancy.

The study was approved by the Local Ethics Committee of the Second Affiliated Hospital of Xinxiang Medical University. Our study was executed in accordance with the Helsinki Declaration. The subjects gave their written informed consent after a complete explanation. This study has been registered in ClinicalTrials.gov (NCT03451734).

### 2.2. Image Acquisition and Processing

A 3.0 T Siemens scanner (Germany) was applied to scan the patients at baseline and after eight weeks of treatment. Healthy controls were scanned once at baseline. Data were preprocessed through the Data Processing Assistant for Resting-State fMRI software. The detailed information of data acquisition and preprocessing is provided in Supplementary Materials (available here).

### 2.3. VMHC Analyses

VMHC was processed using the REST software. The detailed information of VMHC analyses is offered in Supplementary Materials.

### 2.4. Statistical Analyses

The demographic and clinical characteristics were compared by two-sample tests or the chi-square test when necessary. All patients at baseline were compared with healthy controls based on the Generalized Linear Model (GLM) to obtain brain regions with abnormal VMHC with age, framewise displacement, and years of education as covariates, and brain regions with abnormal VMHC were generated as a mask. Then, paired sample *t*-tests based on GLM were applied to compare the differences in VMHC from baseline to eight weeks for the patient group within the brain mask. The Gaussian random field theory was applied to correct for multiple comparisons at *p* < 0.05 by the REST software (voxel significance: *p* < 0.001, cluster significance: *p* < 0.05). Once the apparent differences in VMHC from baseline to eight weeks for the patient group were found, we defined the resultant brain regions as regions of interest (ROIs) and extracted their VMHC values to compare the changes in VMHC between baseline and after eight weeks. The “alterations” of VMHC were that we had subtracted VMHC at baseline from VMHC after eight weeks for the patient group.

To assess the therapeutic effect, the reduction ratio (RR) of the PANSS total scores was calculated as follows:

(1) $$\begin{array}{c} \text{RR}=\frac{{\text{PANSS}}_{\text{total}\_1}-{\text{PANSS}}_{\text{total}\_2}}{{\text{PANSS}}_{\text{total}\_1}}, \end{array}$$

where PANSS_total_1_ and PANSS_total_2_ refer to the total PANSS scores at baseline and after eight weeks of therapy, respectively.

It is similar for the alterations of PANSS positive, negative, and general symptom scores and cognition parameter scores.

### 2.5. Correlation Analyses

Pearson's correlation was used to analyze these correlations between abnormal VMHC at baseline and the PANSS/cognition parameter scores of patients and between VMHC alterations and changes in the PANSS/cognition parameter scores of patients after eight weeks of treatment with a threshold of *p* < 0.05.

### 2.6. Classification Analysis by SVR

SVR was applied to explore the competence of the extracted VMHC values in brain regions for predicting the treatment response through applying the LIBSVM software package (http://www.csie.ntu.edu.tw/~cjlin/libsvm/) in MATLAB. SVR was executed for the extracted VMHC (including baseline levels and alterations of VMHC) and each symptomatic domain (PANSS total scores, positive and negative symptoms, and general symptom subscale scores).

The algorithms and training sets used by SVR are described as follows.

Exploring a multiple regression function *f*(*x*) according to *x* through a sample spectrum is the aim of predicting an expected output characteristic. The SVR equation has been clearly described in some literatures [41, 42], which is summarized as follows [43]:

(2) $$\begin{array}{c} f\left(x\right)=a_{0}+\overset{N}{\underset{ij=1}{\sum }}\left(a_{i}-{a_{i}}_{*}\right)\left\{ø\left(\chi i\right).ø\left(\chi j\right)\right\}+b, \end{array}$$

where 0 ≤ *αi* and *αi*∗≤*C*. *C* is a complementary parameter specifying the regularization constant or penalty error, which defines the trade-off between model simplicity and training error. Parameter *a* is the Lagrange multiplier meeting. Parameters *a* and *C* are detailedly described in the literatures [42, 44]. The capacity to conduct both linear and nonlinear data through the kernel is a valuable characteristic of the SVR. The effectiveness of the optimal model is verified in the process of prediction. In order to optimize the parameters of the SVR model, the cross-validation method is used to search the optimal parameters [41]. The training set is divided into four equally sized subsets, one of which is tested using predictor training in the remaining three subsets in order to find better values of *ε* and *C*. A grid search is performed on a predefined parameter space. Finally, the model with the highest prediction accuracy is adopted (that is, minimum cross-validation error).

## 3. Results

### 3.1. Demographic and Clinical Characteristics of the Subjects

One patient was excluded because of excessive head movement. The final sample included 20 schizophrenic patients and 20 healthy controls. No significant difference was found between the age and sex ratios of the two groups, but the years of education significantly differed (*p* < 0.05). The mean dosage of olanzapine was 20.50 ± 1.54 mg/day (Table 1).

### 3.2. Improvement of Symptoms after Treatment

The PANSS negative, positive, and general symptom subscale scores and total scores of the patient group after eight weeks of olanzapine therapy significantly improved compared with the baseline scores (27.40 ± 5.42 vs. 14.55 ± 5.12, 22.80 ± 5.82 vs. 12.30 ± 3.85, 52.80 ± 5.11 vs. 29.20 ± 5.51, and 103.00 ± 10.79 vs. 56.05 ± 12.08) (*p* ≤ 0.001, Table 2). After eight weeks of olanzapine therapy, the cognitive function tests, including TMT-A, BACS-SC, HVLT-R, WMS-SS, NAB-M, BVMT-R, CF-ANF, MSCIT, and CPT-IP, also became significantly improved compared with those at baseline (55.09 ± 22.11 vs. 33.44 ± 11.54, 37.85 ± 10.79 vs. 44.25 ± 11.02, 17.15 ± 3.79 vs. 22.10 ± 4.09, 11.95 ± 2.65 vs. 15.10 ± 2.94, 9.35 ± 5.66 vs. 15.95 ± 5.71, 18.35 ± 6.62 vs. 27.05 ± 5.81, 13.65 ± 3.22 vs. 17.95 ± 2.26, 79.04 ± 9.19 vs. 90.75 ± 13.28, and 1.03 ± 0.56 vs. 1.80 ± 0.54) (*p* ≤ 0.001, Table 2).

### 3.3. VMHC Results

**Figure 1:**
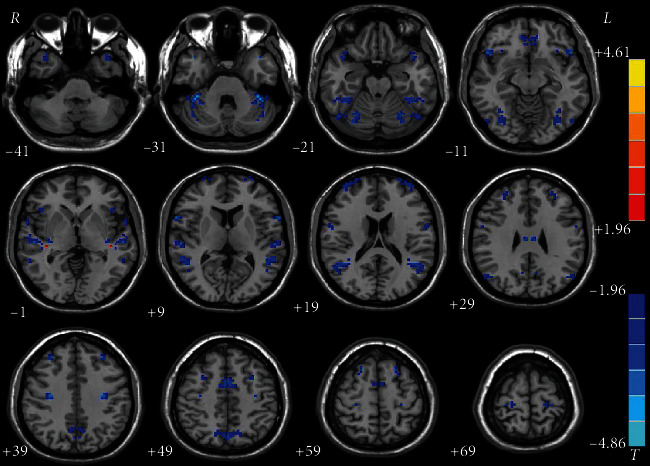
Brain regions with significant difference in VMHC between patients at baseline and healthy controls. Brain regions with significant difference in VMHC were the orbital IFG/STG, fusiform gyrus/cerebellum VI, STG, MTG/angular gyrus, opercular IFG, superior and middle MPFC, superior/middle MPFC, precentral gyrus/postcentral gyrus, precuneus, medial orbital FG, precentral gyrus, and median cingulum gyrus. The color bar represents the *T* values of the group analysis of VMHC. VMHC = voxel-mirrored homotopic connectivity; IFG = inferior frontal gyrus; STG = superior temporal gyrus; MPFC = medial prefrontal cortex; MTG = middle temporal gyrus; FG = frontal gyrus.

**Figure 2:**
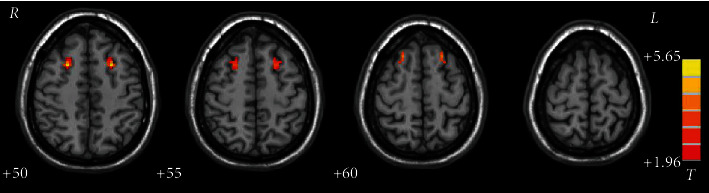
Treatment effects on VMHC between patients at baseline and after eight weeks of treatment. Brain regions with significant increase in VMHC were the superior/middle MPFC. MPFC = medial prefrontal cortex.

The patients had significantly decreased VMHC in the orbital inferior frontal gyrus (IFG)/STG, fusiform gyrus/cerebellum VI, STG, middle temporal gyrus (MTG)/angular gyrus, opercular IFG, superior and middle MPFC, precentral gyrus, superior/middle MPFC, precentral gyrus/postcentral gyrus, precuneus, and medial orbital frontal gyrus (FG) and increased VMHC values in the median cingulum gyrus of patients relative to the controls at baseline (Table 2 and Figure 1 present the detailed information). VMHC in the superior/middle MPFC increased after eight weeks of treatment relative to the baseline. The details are offered in Table 2 and Figure 2.

### 3.4. Correlation Analyses

No correlation was revealed between the abnormal VMHC and PANSS/cognition parameter scores of patients at baseline. No correlation was found between the VMHC alterations and reductions in the PANSS/cognition parameter scores of patients after eight weeks of treatment.

### 3.5. Classification Analysis through SVR

**Figure 3:**
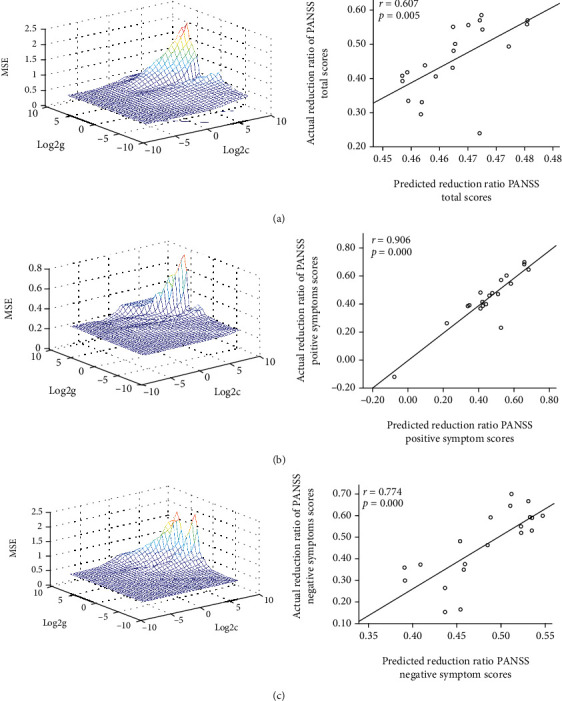
SVR results suggest that high VMHC levels at baseline in the superior/middle MPFC predict therapeutic response in the patient group. (a–c, left) SVR parameter selection results (3D visualization). log2*c* and log2*g* were the logarithm of parameters *c* and *g*. (a–c, right) A positive correlation between predicted and actual RRs of the PANSS total scores (*r* = 0.607, *p* = 0.005), positive symptom subscale scores (*r* = 0.906, *p* ≤ 0.001), and negative symptom subscale scores (*r* = 0.774, *p* ≤ 0.001) of individual patients after eight weeks of olanzapine treatment. VMHC = voxel-mirrored homotopic connectivity; SVR = support vector regression; PANSS = positive and negative syndrome scale; MPFC = medial prefrontal cortex; RR = reduction ratio.

SVR analysis was performed to explore whether the extracted VMHC values in certain brain regions could predict responses to olanzapine treatment in the patients. The SVR results revealed significantly positive correlations between the baseline VMHC in the superior/middle MPFC and the RRs of PANSS total scores (*r* = 0.607, *p* = 0.005) and positive (*r* = 0.906, *p* ≤ 0.001) and negative (*r* = 0.774, *p* ≤ 0.001) symptom subscale scores (Figure 3 presents the details). No significant relationship was found between the alterations in VMHC values in the superior/middle MPFC and the RRs of PANSS total and subscale scores.

## 4. Discussion

The present results revealed that schizophrenic patients had reduced VMHC in the orbital IFG/STG, fusiform gyrus/cerebellum VI, STG, MTG/angular gyrus, opercular IFG, superior and middle MPFC, precentral gyrus, superior/middle MPFC, precentral gyrus/postcentral gyrus, precuneus, and medial orbital FG and increased VMHC in the median cingulum gyrus relative to the controls at baseline. The patients presented increased VMHC in the superior/middle MPFC after eight weeks of treatment. Besides, no correlation was found between the abnormal VMHC and the PANSS/cognition parameter scores for the patients at baseline and between the VMHC changes and the reductions in the PANSS/cognition parameter scores for the patients after treatment. Moreover, SVR analysis revealed that the baseline VMHC in the superior/middle MPFC can predict the symptomatic improvement of PANSS after eight weeks of olanzapine treatment.

Our main findings showed that olanzapine may normalize decreased VMHC in schizophrenic patients, which is consistent with our hypothesis. Schizophrenic patients displayed reduced VMHC in large brain regions at baseline, including TG, IFG, MPFC, and motor and low-level sensory processing regions, which comprised the fusiform gyrus and cerebellum lobule VI. Reduced VMHC in the frontal lobe was normalized by olanzapine treatment. Our previous studies found that patients with recurrent schizophrenia had reduced VMHC in the DMN, including the inferior parietal lobule, precuneus, lingual and fusiform gyri, and cerebellum lobule VI [14]. Hoptman et al. revealed that schizophrenic patients or schizoaffective disorder had reduced VMHC in the thalamus cerebellum and occipital lobe [2]. Meanwhile, significantly low VMHC in the STG/insula, fusiform and precentral gyri, and precuneus have been reported in unmedicated adolescent-onset schizophrenic patients [10]. The baseline results of the present study are consistent with those of the abovementioned studies. Processing information bihemispherically in human hemispheres is beneficial [45], and homotopic interaction may play an important role in the strategic deployment of attention reaction to task requirements [46] and sustained attention [47]. Hence, decreased VMHC provides evidence for the dysconnectivity in homotopic brain regions in schizophrenia. This phenomenon may be related to some of the attention issues observed in schizophrenia.

Olanzapine treatment normalized VMHC in the MPFC after eight weeks of treatment. The frontal cortex has an established influence on the formation of concepts, synthesis of information, mental flexibility, self-awareness, and self-monitoring [48] and is one of the key regions that participated in the pathophysiology of schizophrenia [49]. Frontal hypoconnectivity and hypoactivity have been found in untreated schizophrenic patients [25, 50]. The interaction of abnormal functions between the prefrontal cortex and the broadly distributed brain regions, including regions in the DMN, parietal cortex, and temporal regions, has been found in schizophrenic patients at rest and in performing some cognitive tasks, including continuous performance and memory working tasks [51]. The variability of the patterns of abnormal MPFC connectivity in patients is involved with the severity of cognitive function damage and primary symptoms, including hallucinations [52]. Cortical thicknesses across several frontal regions in both hemispheres are reduced in first-episode schizophrenic patients [53]. A review revealed that the changes in the resting-state functional connectivity in frontal regions seem to be in the direction of normalization after antipsychotic therapy when control data are accessible. The alterations observed with antipsychotic treatment are at all times augmented in brain region activation or functional connectivity when control data are unavailable, suggesting that the regulation of the frontal region function is a critical mechanism [54]. Hence, the effects of normalization on VMHC in the frontal cortex may be associated with the benefit of olanzapine therapy.

The SVR results have revealed that the high VMHC values in the MPFC at baseline may predict the positive or negative symptom improvement of PANSS after eight weeks of olanzapine treatment. The precuneus, frontal orbital cortex, and dorsal MPFC are associated with predicting the clinical outcome in posttraumatic stress disorder via fMRI [55]. The hemodynamic activation of the frontotemporal region may predict therapeutic reaction to selective serotonin reuptake inhibitors in MDD [56]. Jiang et al. showed that gray matter of the precuneus and left postcentral gyrus was related to the predictions for electroconvulsive therapy response [57]. The present results revealed that increased VMHC in the superior/middle MPFC can predict clinical therapy response, highlighting the importance of the frontal region activity in antipsychotic treatment, and may account for clinical symptomatic improvement from functional brain imaging.

Previous studies have revealed associations between abnormal brain neural activity and symptom severity in schizophrenia [1, 11]. And we hypothesized that abnormal VMHC values or alterations were correlated with clinical variables in the patients. However, no correlations were found between abnormal VMHC and PANSS/cognition parameter scores of patients at baseline. There were also no correlations between the VMHC alterations and reductions in the PANSS/cognition parameter scores of patients after eight weeks of treatment. One possibility for no correlations was that abnormal VMHC might be a trait alteration for the patients with schizophrenia independent of the clinical symptom severity and illness duration. Another possibility might be a relatively small sample size in the present study.

Several limitations should be considered. First, the sample size was small, which could greatly inflate the false-positive discoveries in the observational data. However, our study had a responsible criterion for inclusion, and some previous longitudinal studies also showed similar sample size [58–61]. With an observational design and in an exploratory or discovery framework, a much bigger sample would be necessary to avoid false discovery. Second, white/gray matter volume abnormalities were not evaluated. Therefore, whether these volume abnormalities caused changes in VMHC remains unclear. Third, because the control group was scanned only at baseline in the present study, a repeated-measure comparison with changes in the control group was absent. Hence, we could not fully attribute these changes to the intervention. However, previous studies have revealed that VMHC is a relatively stable resting-state fMRI metric [62]. Intraindividual fluctuations between baseline (t1) and final scans (t2) on the fMRI measures might be limited in healthy controls. Fourth, previous antipsychotics for the relapsing patients were not recorded. Fifth, a symmetrical standard template was used, and functional images were smoothed to improve the functional coordination between mirrored regions. Given that the human brain is not symmetrical, the effects of brain asymmetry cannot be eliminated although reduced VMHC cannot be interpreted for morphometric asymmetry.

In conclusion, our findings show that olanzapine may enhance VMHC in the superior/middle MPFC, which may predict therapeutic response in clinical symptoms to antipsychotic therapy in schizophrenic patients. Hence, our preliminary findings conduce to the comprehension of the therapy effects of antipsychotic medications on homotopic connectivity in schizophrenia. Of note, as the researches of Chen et al. [63] and Turner et al. [64] suggested, small sample sizes would reduce the reliability and replicability of the results. Hence, a future expanded version of the article should be necessary to give more consistency to the initial results.

## Figures and Tables

**Table 1:** Characteristics of the subjects.

	Patients	Controls	*χ*^2^/*T*	*p* value
Sex (male/female)	15/5	14/6	0.125	0.723^a^
Age (years)	22.75 ± 4.38	25.70 ± 4.90	-2.008	0.052^b^
Years of education (years)	10.65 ± 2.50	12.75 ± 2.95	-2.428	0.020^b^
Dose of olanzapine (mg/day)	20.50 ± 1.54			
At baseline				
PANSS	103.00 ± 10.79			
Positive	22.80 ± 5.82			
Negative	27.40 ± 5.42			
General	52.80 ± 5.11			
TMT-A	55.09 ± 22.11			
BACS-SC	37.85 ± 10.79			
HVLT-R	17.15 ± 3.79			
WMS-SS	11.95 ± 2.65			
NAB-M	9.35 ± 5.66			
BVMT-R	18.35 ± 6.62			
CF-ANF	13.65 ± 3.22			
MSCIT	79.04 ± 9.19			
CPT-IP	1.03 ± 0.56			
After 8 weeks of treatment				
PANSS	56.05 ± 12.08			
Positive	12.30 ± 3.85			
Negative	14.55 ± 5.12			
General	29.20 ± 5.51			
TMT-A	33.44 ± 11.54			
BACS-SC	44.25 ± 11.02			
HVLT-R	22.1 ± 4.09			
WMS-SS	15.1 ± 2.94			
NAB-M	15.95 ± 5.71			
BVMT-R	27.05 ± 5.81			
CF-ANF	17.95 ± 2.26			
MSCIT	90.75 ± 13.28			
CPT-IP	1.80 ± 0.54			

^a^The *p* values were obtained by the chi-square test. ^b^The *p* values were obtained by two-sample *t*-tests. PANSS = positive and negative syndrome scale; TMT-A = Trail Making Test, part A; BACS-SC = Brief Assessment of Cognition in Schizophrenia Symbol Coding Test; HVLT-R = Hopkins Verbal Learning Test-Revised; NAB-M = Neuropsychological Assessment Battery-Mazes; WMS-SS = Wechsler Memory Scale Spatial Span; CF-ANF = Category Fluency-Animal Naming Fluency; BVMT-R = Brief Visuospatial Memory Test-Revised; MSCIT = Mayer-Salovey-Caruso Emotional Intelligence Test; CPT-IP = Continuous Performance Test-Identical Pairs.

**Table 2:** Alterations of VMHC across patients (at baseline, after 8 weeks of treatment) and controls.

Cluster location	Peak coordinate			Cluster (voxel)	*T* value
	*x*	*y*	*Z*		
Patients at baseline vs. controls					
Orbital IFG/STG	±51	21	-12	184	-3.6668
Fusiform gyrus/cerebellum VI	±39	-42	-30	392	-3.9664
STG	±54	-21	0	118	-3.7539
MTG/angular gyrus	±45	-51	18	200	-3.3522
Opercular IFG	±63	12	9	84	-3.4363
Superior MPFC	±27	66	15	70	-3.4842
Middle MPFC	±30	39	39	64	-3.6280
Precentral gyrus	±33	-15	36	66	-3.8381
Superior/middle MPFC	±21	15	54	92	-3.2115
Precentral gyrus/postcentral gyrus	±36	-27	66	60	-2.8298
Precuneus	±0	-75	48	200	-3.0148
Median cingulum gyrus	±0	-18	36	118	3.1627
Medial orbital FG	±0	39	-12	66	-2.5950
Patients after 8 weeks vs. at baseline					
Superior/middle MPFC	±24	12	51	62	5.6466

VMHC = voxel-mirrored homotopic connectivity; IFG = inferior frontal gyrus; STG = superior temporal gyrus; MPFC = medial prefrontal cortex; MTG = middle temporal gyrus; FG = frontal gyrus.

## Data Availability

All data included in this study are available upon request by contact with the corresponding author.

## Supplementary Materials

Supplementary Materials The detailed information of data acquisition and preprocessing is provided in the supplemental file.
