# Supplementary material for: Increased Homotopic Connectivity in the Prefrontal Cortex Modulated by Olanzapine Predicts Therapeutic Efficacy in Patients with Schizophrenia
Source: Neural Plast. 2021 Sep 1;2021:9954547. doi: 10.1155/2021/9954547 (PMC8429031; doi:10.1155/2021/9954547)
Supplement: Supplementary Materials — The detailed information of data acquisition and preprocessing is provided in the supplemental file. [file 9954547.f1.docx]

**Supplemental Methods**

**Data acquisition**

Scanner parameters were as follows: repetition time/echo time = 2000/30 ms, scan time = 8 minutes, 33 axial slices, 64×64 matrix, 90° ﬂip angle, 22 cm ﬁeld of view, 4 mm section thickness, 0.6 mm slice gap, and 240 volumes. All participants were required to lie on the scanner with their eyes closed. They used soundproof headphones and remained still.

**Data preprocessing**

The resting state fMRI data were preprocessed using the DPARSF software. The ﬁrst 10 images were excluded from analysis due to the instability of the initial MRI signal and for the participants to adapt to circumstances. Afterwards, slice timing and head motion correction were conducted. All subjects had no more than 2 mm of translation in the x, y, or z axis and 2° of rotation in each axis. The imaging data were then spatially normalized to the standard MNI EPI template in SPM8 instead of individual 3D T1-weighted images and resampled to 3 mm×3 mm×3 mm and smoothed with Gaussian kernel (full width at half maximum: 4 mm). Finally, the imaging data were linearly detrended and temporally band-pass-filtered (0.01–0.08 Hz) to decrease the effect of low-frequency drifts and physiological high-frequency noise. Spurious covariates, including signal from the white matter centered region and ventricular ROI, as well as the 24 head motion parameters obtained by rigid body correction, were removed. Scrubbing (removing time points with FD > 0.2mm) was also applied as an aggressive head motion control strategy.

**VMHC analyses**

For each subject, Pearson correlations were calculated between a given voxel time series and that of its opposite hemisphere to generate the homotopic functional connectivity. The Fisher z-transformed correlation coefﬁcients were applied to improve normality. Then, the VMHC maps were generated.
